# Declining bacteria, lead, and sulphate, and rising pH and oxygen in the lower Mississippi River

**DOI:** 10.1007/s13280-020-01499-2

**Published:** 2021-02-07

**Authors:** R. Eugene Turner

**Affiliations:** Department of Oceanography and Coastal Sciences, Louisiana State University, Baton Rouge, LA 70803 USA

**Keywords:** Historical, Mississippi River, Monitoring, Policy, Pollution, Water quality

## Abstract

Various air and water pollution issues in the US were confronted in the last 60 years using national policy legislation, notably the Clean Water Act and the Clean Air Act. I examine changes in the concentrations of bacteria, oxygen, lead, and sulphate at the terminus of the Mississippi River before and after these pollution abatement efforts. Microbial concentrations increased or were stable from 1909 to 1980 but decreased about 3 orders of magnitude after the 1970s, while the average oxygen content increased. A large decline in lead concentration occurred after the 1960s, along with a less dramatic decline in sulphate concentrations. The pH of the river dropped to a low of 5.8 in 1965 as sulfur dioxide emissions peaked and averaged 8.2 in 2019 after emissions declined. Decades of efforts at a national scale created water quality improvements and are an example for addressing new and existing water quality challenges.

**Supplementary Information:**

The online version of this article (10.1007/s13280-020-01499-2) contains supplementary material, which is available to authorized users.

## Introduction

Waterways in the early 1900s were a drinking water source and also a direct recipient of many kinds of wastes. It was then that the earliest surveys of river chemistry were done as understanding developed about the nexus between water quality and health (Palmer 1903; Dole 1909; Stets et al. 2012). Streeter and Phelps (1925), sanitary engineers, demonstrated the ‘generality of laws’ governing oxygen uptake and reaeration in the ‘natural purification’ of the Ohio River. Various forms of nitrogen, phosphorus, salts, metals, and sediments in riverine fluxes increased in the US and throughout the world in the last 100 years (Nriagu and Pacyna 1988; Meybeck 2003; Syvitski et al. 2003) while, simultaneously, nascent intentions to minimize further change or to restore water quality permeated societies, principally to protect health (Webb 2020), but also agriculture, fisheries, recreation and public well-being. The choices for action, like today, were not without disagreement. The Thames in the 1800s, for example, was “hissing like soda water with baneful gases” and carried diseases like typhoid and cholera that killed tens of thousands each year (Schneer 2015), yet a 1854 writer to the Times (London) complained that “We prefer to take our chance of cholera and the rest (rather) than be bullied into health.” (Lacey 2007, p. 396). But river restoration did occur. The oxygen concentration in the lower Thames River was zero in summers up to the 1960s but was restored to 50% saturation by 1980 and had re-established salmon migrations (Andrews and Rickard 1984). The July–August oxygen content in the Delaware River near Philadelphia went from near 100% saturation in 1880 to zero in the 1950s to early 1960s, and then recovered to around 75% in the first decade of this century (Sharp 2010). These granular improvements in water quality reflected a growing concern about there being greater risks in the last century from chronic disease than war (Li et al. 2020).

In the United States, the Clean Water Act (CWA; 1972) established a national structure to reduce pollutant discharges into water and the Toxic Substances Control Act of 1976 and other acts provide the US Environmental Protection Agency (EPA) authority to restrict chemical amounts and uses, including lead, a well-known toxin in paint and gasoline, particularly for children (Gould 2009). The Air Pollution Control Act of 1955 was the first Federal legislation involving air pollution and was followed by the Clean Air Act (CAA) of 1963 and significant amendments in 1970 and 1990 establishing enforced national air quality standards with a focus on acid rain (pH < 5) that affects terrestrial and aquatic ecosystems (Cogbill and Likens 1974; Burton and Aherne 2012). Management of acidic precipitation was primarily concerned with reducing sulfur and nitrogen emissions when the CAA was passed in 1970 (Likens et al. 1996). These and other State and Federal laws and regulations attempted to improve air and water quality and reduce toxic exposures, principally from a health perspective. Indeed, the CWA was formed to address the various ‘externalities’ of water pollution for those downstream of sewage plants dumping waste into the river, or industrial wastes such as caused the Cuyahoga River to catch fire in 1969. The magazine Time (1969) dryly described the river then: “Anyone who falls into the Cuyahoga does not drown—he decays.”

But sometimes policies have unforeseen consequences, and the tensions between public goods and private uses can become unexpectedly relaxed or heightened as policies are implemented. The social contract upon which governance rests is informed by data to determine whether to abandon or modify failures, initiate new approaches, and celebrate successes. One might ask, for example, if air quality changes are reflected in water quality—are water quality improvements representative of local or regional policy? Or, how soon after policy implementation did stream condition change and were changes sustained? Here I examine a few key water quality changes at the terminus of the Mississippi River as it enters the Gulf of Mexico from before to after the CWA was passed. The focus is on pH and the concentrations of bacteria, oxygen, lead, and sulphate that are influenced by well-known landscape, airscape and point-source influences. I used archived water quality data collected from 1901 to 2019 by Federal, State, and the New Orleans Sewerage and Water Board (NOSWB) to reconstruct a 100+ year record of some key indicators of water quality in the lower Mississippi River.

The Mississippi River, the largest river in North America, drains 41% of the US and parts of Canada and brings 80% of the water, and 91% and 88% of the nitrogen and phosphorus, respectively, entering the Gulf of Mexico from the US (Dunn 1996). Land cover in the 2 980 000 km^2^ large watershed is about 58% cropland, 21% pasture, 18% barren, 2.4% forest, and 0.6% wetland and urban (Goolsby et al. 1999) and it has a population of about 30 million people. It also has many farm animals producing much waste. Iowa, for example, nearly 10% of the area, has a population of 2.8 million people, had 6.2 million bovine, 25 million pigs, 12 million turkeys, and 56 million chickens in 2019 (https://www.nass.usda.gov/Quick_Stats/Ag_Overview/stateOverview.php?state=IOWA). The watershed was not part of the excellent summary by Lajtha and Jones (2013) on the various differences in air quality in Europe and the US after the CAA was passed, primarily because of either an absence of suitable air quality data or an emphasis on the areas thought to have more severe acid rain impacts. Published reviews of the connections between air and water quality at the end of this river are apparently non-existent or sparse.

## Materials and Methods

### Water quality archival data

**Fig. 1:**
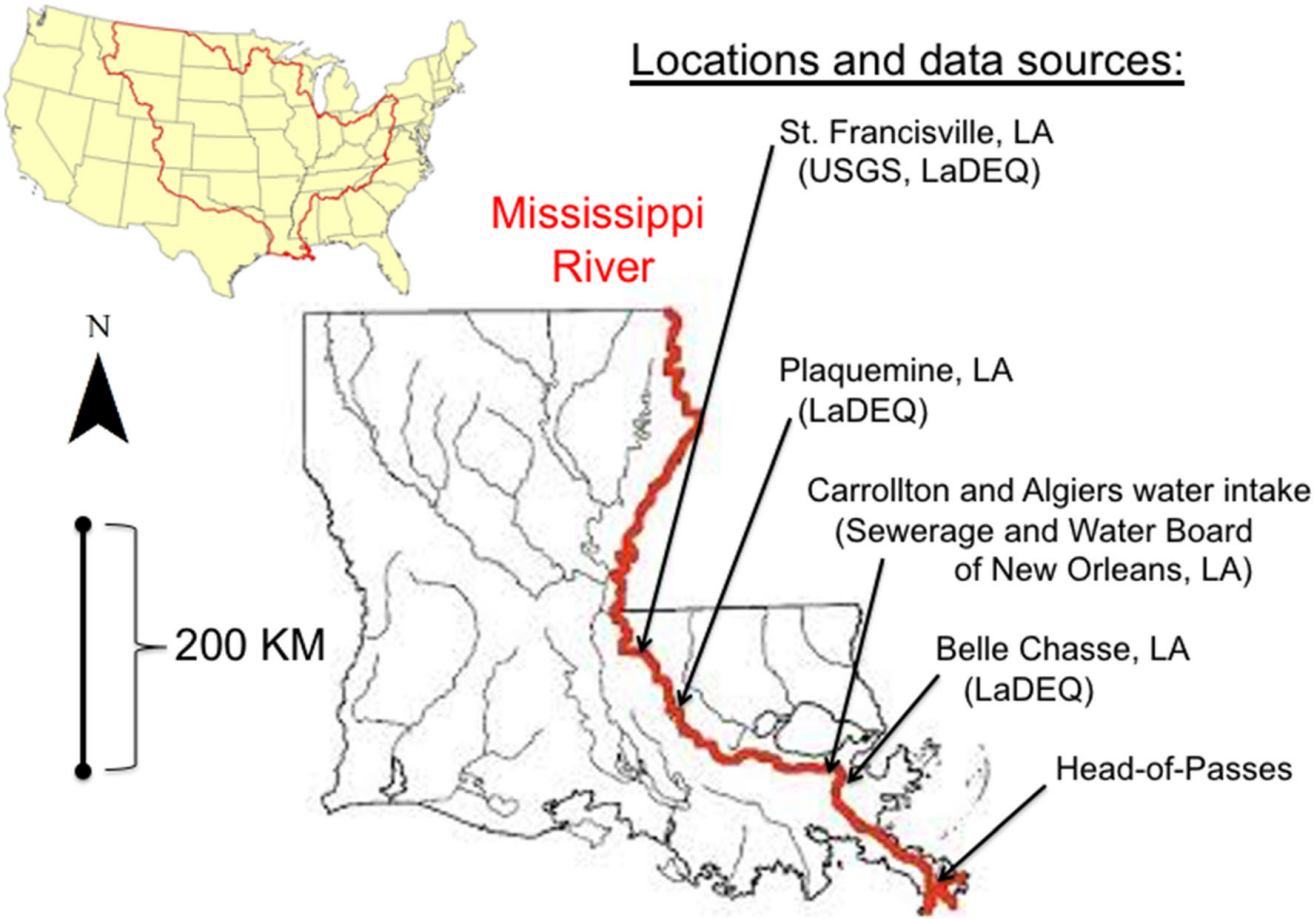
Location map of sampling stations and data sources. The Mississippi River watershed is outlined in the map of the United States in the upper left. The Mississippi River sampling stations are at: (1) St. Francisville, LA (United States Geological Survey; USGS) and Louisiana Department of Environmental Quality; LaDEQ); (2) Plaquemine, LA (LaDEQ); (3) New Orleans, LA (New Orleans Sewerage and Water Board sampling at Carrollton and Algiers water intakes; NOSWB); (4) Belle Chasse, LA (LaDEQ)

Water quality data are from four locations on the southern end of the Mississippi River at St. Francisville, Plaquemine, two locations in New Orleans, and at Belle Chasse, LA, located 428, 335, 167–153 and 122 km upstream, respectively, from the Head-of-Passes where the river divides into three main outlets to the Gulf of Mexico (Fig. 1). The two major metropolitan areas between St. Francisville and the Gulf of Mexico are Baton Rouge and New Orleans (ca. 830 thousand and 1270 thousand population size, respectively, in 2020).

The Louisiana Department of Environmental Quality (LaDEQ) sampling data are from 1978 to 2018 and occur at three locations: St. Francisville stations 9, 55, 318, and 4031, Plaquemine stations 53, 54 and 319, and Belle Chase stations 51, 52, and 320. The Plaquemine and Belle Chasse stations are associated with a ferry crossing. The LaDEQ stations were sampled for lead, sulphate and bacteria as early as 1966 and are continuing, but they are not complete for 2019. Fifty-five lead values were removed from the data set consisting of eighteen consecutive lead values of 5.0 µg L^−1^ for all samples collected from 1998 to 2000 for each of the three locations in the LaDEQ data set. An additional one from the December 1997 Plaquemine data set was also removed. There were only 2 to 5 lead data reported annually from 2001 to 2011 for the LaDEQ data, after which there are no data reported. The 1980 sulphate data consisted of only 3 annual measurements and was not used. All other sulphate sampling had at least 9 data points annually (range 9–36 year^−1^). Other values removed from the data set were data entry errors indicated by negative values.

The New Orleans samples for bacteria are from river water intake pipes at the Carrollton and Algiers water treatment plants that bring drinking water to the metropolitan New Orleans area. The NOSWB has used the same water intake location in regular service since December 1900 (Sewerage and Water Board 1903), and data were copied from annual reports in their offices. The NOWSB counted bacterial colonies on agar plates incubated for 24 h at 37 °C from 1908 to 1955. They are counts of bacteria that can grow on the agar media and are, therefore, not direct counts determined through epifluorescence. The total bacteria counts appear to have been made daily, but not all records survived for inspection. These agar plate measurements were made to compare ‘total bacteria’ in the river to the drinking water supplied and are reported as monthly averages. The annual average used here is the mean ± 1 SE for the 12 months. Agar plate measurements were replaced with estimates of the total coliform bacteria density reported in annual reports (*n* = 139 to 382 year^−1^) but are not available for all years. The LaDEQ results used a membrane filtration method (code SM9222E; https://www.epa.gov/sites/production/files/2017-02/documents/gwr_approved_methods.pdf). Not all coliforms are fecal coliforms and method choices may affect number counts. The fecal coliform data from 1968 to 2018 for the LaDEQ stations at each location were summarized by month and the median value (µ ± 1 SE) for the 12 months.

Other bacteria densities were made by the United States Geological Survey (USGS) at St. Francisville, La (https://nwis.waterdata.usgs.gov/usa/nwis/qwdata/?site_no=07373420; parameter code 31625) that uses a 0.7-µm filter and m-ENDO media. The USGS data has estimates of total coliform and fecal coliform densities for before the LaDEQ data collections started in 1968 and overlap with the often more numerous annual collections by LaDEQ. Only years with 3 or more samples were included.

The combined data set has three different kinds of bacterial density measurements for Mississippi River water. The average density is shown for: (1) the total coliform and fecal coliform density at St. Francisville (USGS; 1969 to 1999 and 1969 to 2008, respectively); (2) the average of the fecal coliform density measurements at St. Francisville, Plaquemine, and Belle Chase (1968 to 2018; LaDEQ); (3) estimates of average annual total bacteria in New Orleans (1908 to 1955; NOSWB); (4) average annual total coliform bacteria (1953 to 1993) at New Orleans (NOSWB; 1953 to 1993); and (5) fecal coliform bacteria at Belle Chasse (1978 to 2018; LaDEQ). The data are in the Supplemental Table S1.

The sulphate and pH concentrations are from this same USGS data collection effort and downloaded from https://www.epa.gov/waterdata/water-quality-data-wqx#legacy. The pH measurements are for years with more than 6 samples in 1 year. Individual pH measurements were converted to their antilogs, the antilogs averaged, and then the averages transformed back into a pH value. A 3-year rolling average was computed and graphed together with the minimum and maximum for each year.

### Air quality archival data

The 1990 to 2019 sulfur dioxide emissions for the US are from U.S. Environmental Protection Agency (EPA), Air Emissions Inventories, *Air Pollutant Emissions Trends Data*, National Annual Emissions, available at https://www.epa.gov/air-emissions-inventories/air-pollutant-emissions-trends-data on May 8, 2020. Data from 1900 to 1990 is from No. HS-28. National Air Pollutant Emissions: 1900 to 2000 (U.S. Census Bureau, Statistical Abstract of the United States: 2003, Washington, DC; https://www2.census.gov/library/publications/2004/compendia/statab/123ed/hist/hs-28.pdf?). This website also has national estimates of lead emissions from 1970 to 2000. The average lead concentration in air (µg m^−3^) at seven air quality stations from 1980 to 2019 are from the EPA (https://www.epa.gov/air-trends/lead-trends).

### Statistics

The combined data set has concentrations for bacteria, oxygen, sulphate and lead. Some samplings resulted in multiple data collections for each month and some had only one monthly sample. The values for 1 month were combined, if available, and an average value for the 12 months made to determine an annual average and standard error for that year. I used Prism 8.0c software © 2020 (GraphPad Software, Inc., La Jolla, CA) for statistical analyses. Log transforms were made for graphing purposes.

## Results

### Bacteria

**Fig. 2:**
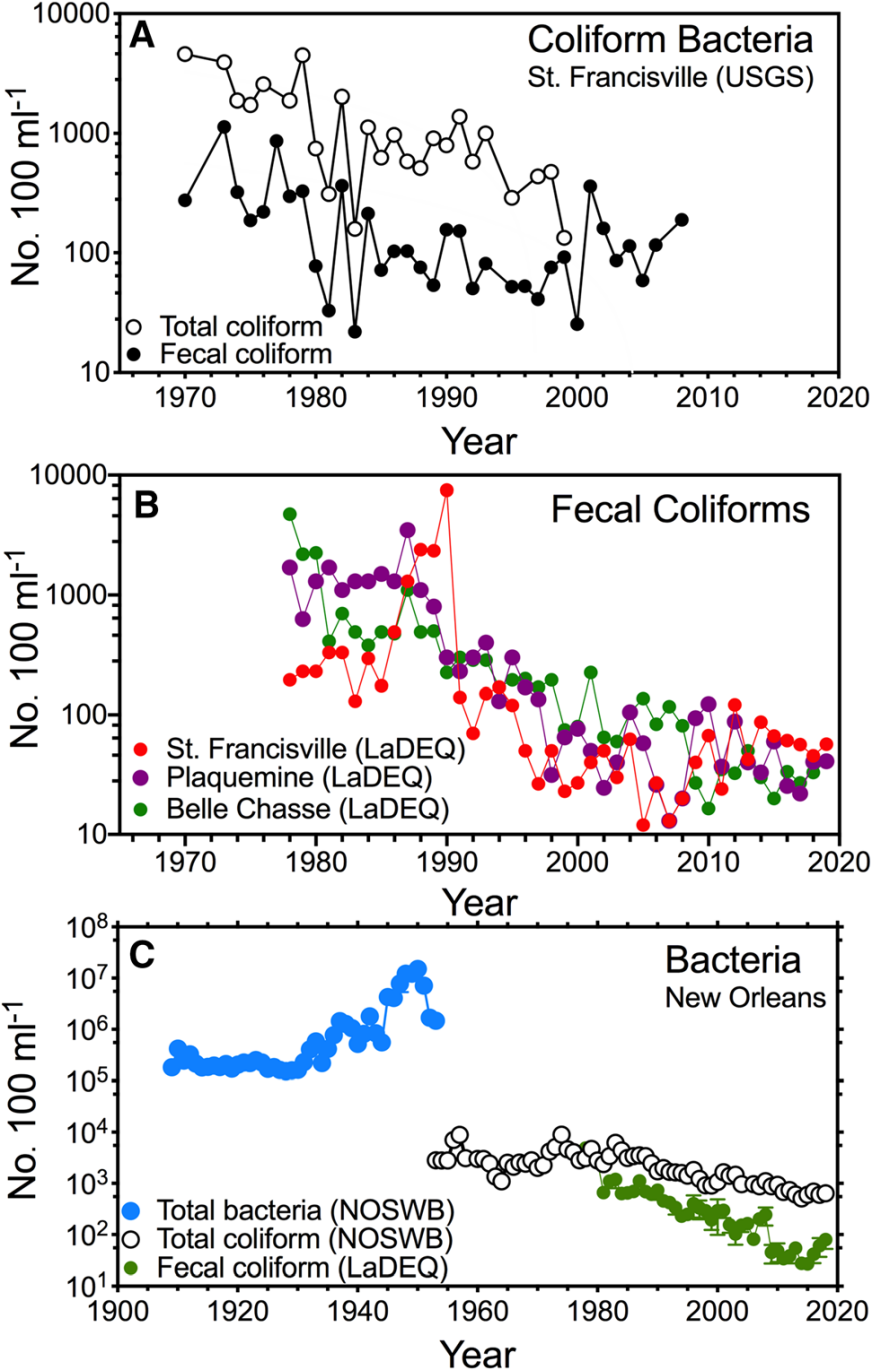
The annual bacterial densities in the lower Mississippi River. **a** Total coliforms and fecal coliform densities at St. Francisville (USGS). **b** Median densities of fecal coliforms (most probable number per 100 ml) measured at St. Francisville, Plaquemine and Belle Chasse (LaDEQ). **c** Three kinds of bacterial densities in the Mississippi River in the New Orleans area from 1909 to 2018. Blue dots are average densities of total bacteria grown on agar plates (NOSWB). White dots are membrane filtration counts of the total coliform bacteria (NOSWB). Green dots are the average fecal coliform bacteria (*n *= 3) at St. Francisville, Plaquemine and Belle Chasse (LaDEQ). The standard error of the mean is shown when individual station numbers are available

The median density of coliform and fecal coliform bacteria declined by more than 2 orders of magnitude after the late 1970s (Fig. 2a and b) until 2010, and were about equal in the last decade, when fecal coliform bacteria densities were below 100 per 100 mL^−1^. The concentrations were lower upstream when compared to downstream except in the 1980s when there was an unexplained rise in the density of fecal coliforms at St. Francisville, followed by a sudden drop in 1990 at all three LaDEQ sampling locations (Fig. 2b). A longer-term record of three different kinds of bacteria density measurements (Fig. 2c) indicates that there was an increase in total bacteria from about 1938 to 1950. The microbiological analyses ceased using agar plates after 1954. The total coliform bacteria density assays used after then yielded relatively stable numbers until the 1980s when they decreased. The fecal coliform densities declined two orders of magnitude since the 1980s.

### Oxygen

**Fig. 3:**
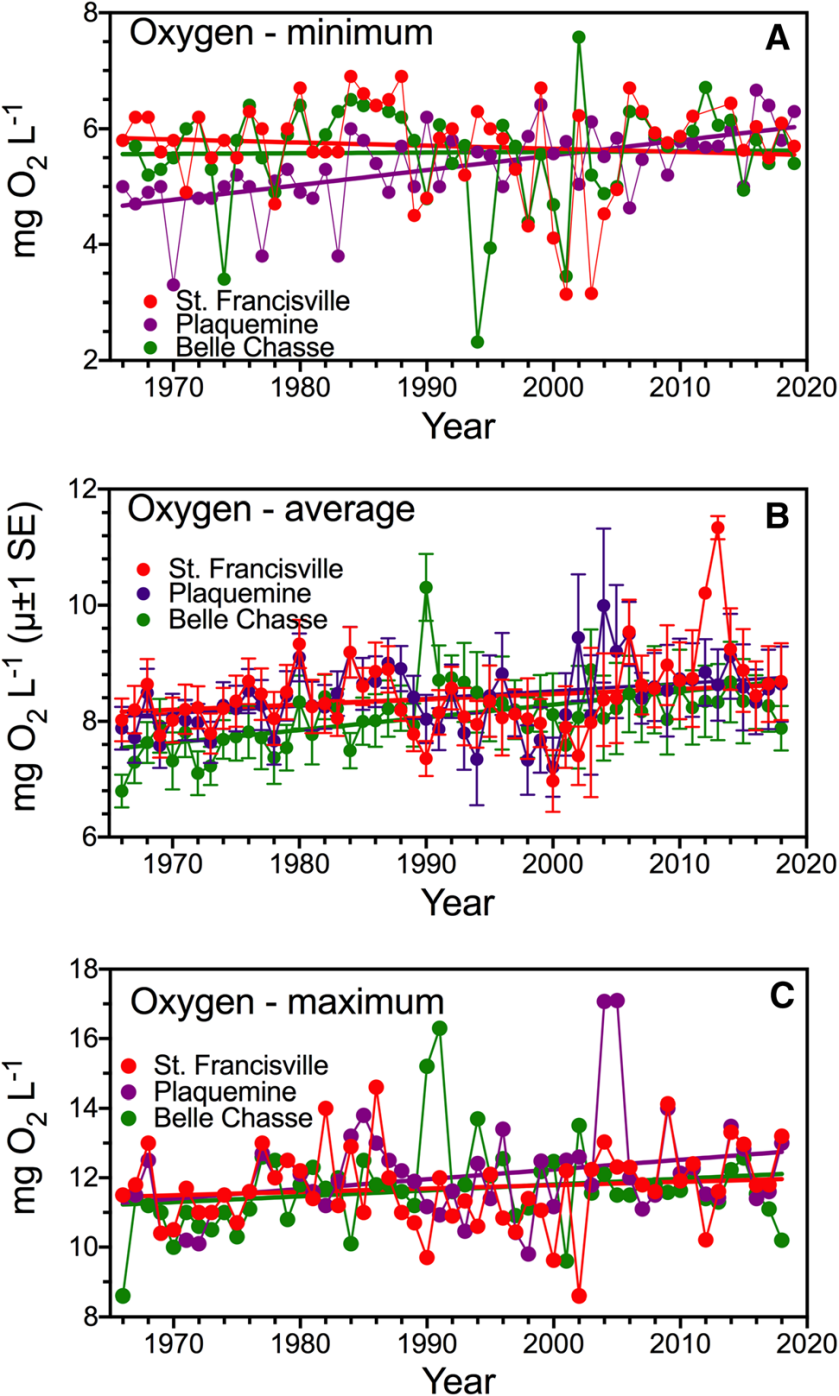
Monthly oxygen concentrations at St. Francisville, Plaquemine and Belle Chasse from 1969 to 2018. **a** Minimum of the monthly values in each year. **b** Average of the monthly values in each year (µ ± 1 SEM). **c** Maximum of the monthly values in each year. Data are from LaDEQ

The lowest concentration of oxygen in a year versus year was not significant at St. Francisville or Plaquemine (Fig. 3a) but had a positive linear slope at Belle Chasse (*F* = 32.7, DFd = 52, *P *< 0.001). The slope of the annual average oxygen concentration versus year was not significant at St. Francisville but had a positive linear slope at both Plaquemine (*F* = 7.52, DFd = 891, *P *< 0.01) and at Belle Chasse (*F* = 22.72, DFd = 955, *P* < 0.0001) (Fig. 3b); the intercepts were different from each other (*F* = 91.60. DFd = 2718, *P *< 0.0001). The slope of the maximum oxygen concentration in a year versus year (Fig. 3c) was stable at St. Francisville and Belle Chasse (*F* = 0.70, DFd = 51, *P* = 0.291) and the intercepts were not different from each other (*F* = 1.243, DFd = 51, *P *= 0.50). The maximum oxygen concentration increased from 1968 to 2018 at Plaquemine (*F* = 7.52, DFd = 51, *P* < 0.01). The summary of the 53 year-long oxygen record is that, on average, oxygen concentrations (minimum, maximum and average) were not different among years at St. Francisville, but the average annual oxygen concentration increased downstream at Plaquemine and Belle Chase, and the maximum oxygen concentration increased at Plaquemine.

### Lead

**Fig. 4:**
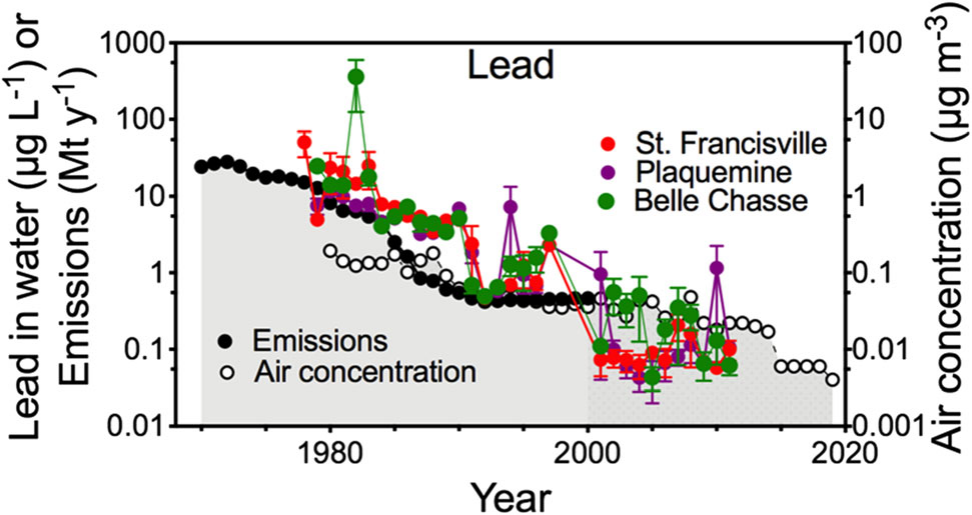
The annual average of monthly concentrations of lead in water (µg L^−1^, µ ± 1 SE) at St. Francisville, Plaquemine, and Belle Chasse from 1969 to 2018 (LaDEQ), and the national estimates of lead emissions (Mt year^−1^) and annual air concentration of lead (µg m^−3^). Note the log scale of both vertical axes

The lead emissions decreased dramatically and are plotted on a log scale along with data on the lead concentrations in water made by the LaDEQ. Lead concentrations in the river declined almost three orders of magnitude from 1979 to 2011 at all three LaDEQ locations (Fig. 4). The concentrations in 1 year were similar at all three sites, indicating that, downstream of St. Francisville, there were no significant inputs of lead into the river or losses from sedimentation. The concentrations in the river stabilized at 0.18 µg Pb L^−1^ from 2001 to 2011, after which sampling stopped. The highest value (in 1982) was more than 2000 times the lowest value (in 2005). The estimated emissions of lead and air concentrations declined from 1980 to 2000, and a linear regression of the two had an R^2^ of 0.48 (not shown).

### Sulphate and pH

**Fig. 5:**
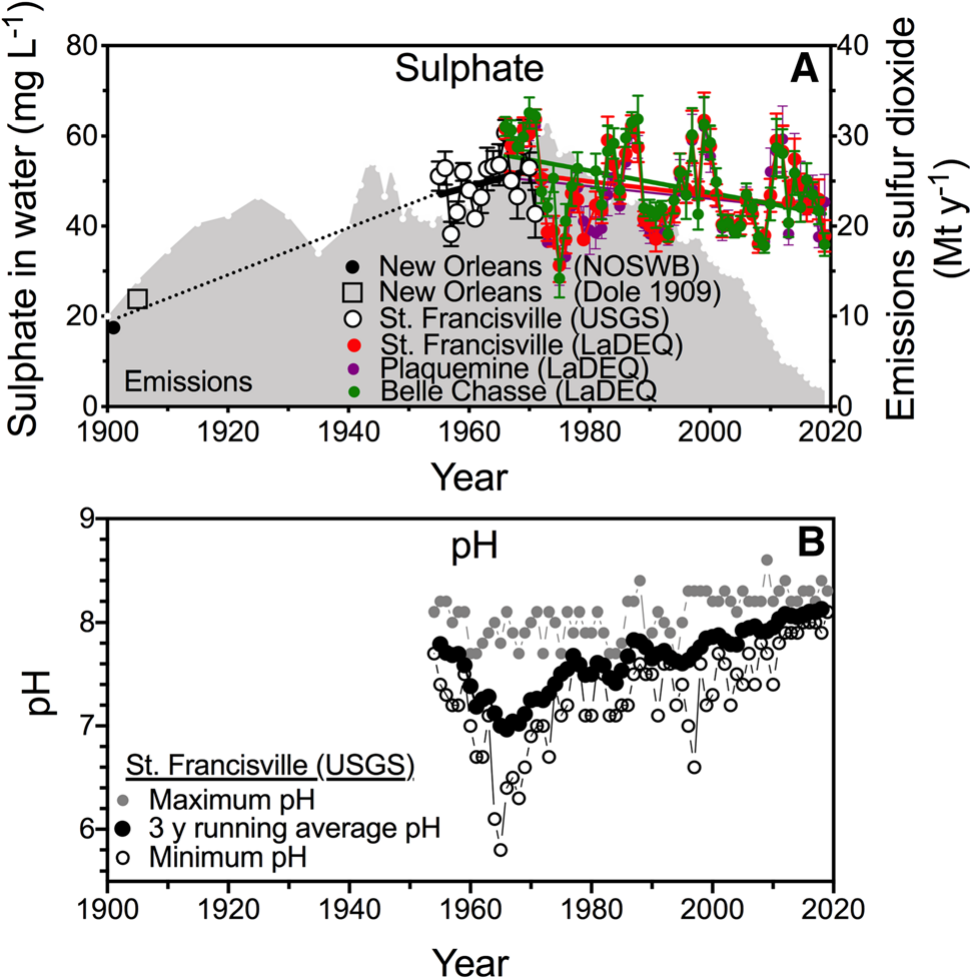
Sulphate (mg L^−1^, µ ± 1 SE) and pH in the lower Mississippi River from 1900 to 2018. A. Sulphate. A dotted line connects values for the beginning of the 20th century to the 1960s. The slopes of the four simple linear regressions are significant (*p* = 0.05 or less), and there is no difference in the slopes for the three LaDEQ stations at St. Francisville, Plaquemine and Belle Chasse. The gray area is the sulphur dioxide emissions data for the USA. B. A 3 year running average of the annual pH from 1954 to 2018 is the black dot, bounded by the minimum and maximum pH for that year

The sulfate concentration in the river was about 20 mg SO_4_ L^−1^ around 1900–1910, and > 50 mg SO_4_ L^−1^ in the 1960s (Fig. 5). The sulphate concentration is now declining slowly relative to the rate of decline in sulfur dioxide emissions, and at insignificantly different rates from 1981 to 2019 (the pooled slope equals − 0.18 mg L year^−1^; *F* = 2.15, DFd = 2707, *P *= 0.07). Figure 5b is a 3-year running average of the pH at St. Francisville, together with the minimum and maximum value for individual years. The annual average pH was 7.8 in 1954, fell to 6.7 in 1965, and then began rising to an average pH of 8.2 in 2019. The lowest value was 5.8 in 1965. The average pH from 1954 to 2019 is a mirror image of the temporal changes in sulfur dioxide emissions shown in Fig. 5a.

## Discussion

The success of the CWA and CAA and amendments is evident in five ways in this data review of water quality in the lower Mississippi River: (1) indicators of microbial concentrations that were increasing or stable from 1909 to 1980, decreased about 3 orders of magnitude after the 1970s; (2) there was a similar large decline in lead concentration after the 1960s, although the routine measurements are no longer reported after 2012; (3) there was a less dramatic, but significant increase in the average oxygen content at the same time. Importantly, the minimum oxygen value was highest at Belle Chasse in the last 2 decades; (4) the sulphate concentrations are declining after emissions dropped, but at a much slower rate than sulfur dioxide emissions. A delayed response (declining sulphate concentrations) has been tied to the rapid loss of calcium and magnesium in soils which recover slowly in the northeast US (Likens et al. 1996), however the concentrations of these two elements appear to have not changed in the Mississippi River (Supplemental Fig. S1); and, (5) the average pH of the river did, in fact, drop with increased sulfur dioxide emissions and has since risen to a pH of 8.2. Thus, emission controls and changes in economic activity reduced SO_2_ emissions (Hedin et al. 1994; Smith et al. 2011) and this had an effect on river pH, but a slower decline in the river’s sulphate content. All of these changes occurred over decades; they were not accomplished quickly after a few masterly reconfigurations of technology or rules, but through sustained attention at many locations, one smokestack or sewerage plant at a time.

George Perkins Marsh published his informed, wide-ranging and, at the time, provocative ‘The Earth as Modified by Human Action’ (Marsh 1885) after traveling widely as a linguist fluent in 20 languages. His observations were not about the first transformation in the Earth’s history, but the one humans were accomplishing so thoroughly, so globally and distinctively that it is now recognized as the Anthropocene. River water quality over the next 100 years was affected as the alarm bells of society went off, legislative initiatives were funded, and new ones infused with authority. The threats to water quality were understood to affect all segments of the population and distinguished by unsubtle causal links to a contamination source. Resistance based on private economic reasons were subsumed as public funding grew and national standards were enforced. Was it a successful effort? Yes, it was, if measured as the concentrations of lead, sulphate, and bacteria in the river declining, the pH becoming less acidic, or oxygen concentrations increasing.

But there are also new agents of often unknown effects added to waterways and their stocks and transformations need monitoring, minimization, and even prohibitive actions. Two well-known ones are the rising alkalinity and nitrate concentrations of the last 100 years (Turner and Rabalais 2003; Raymond et al. 2008). Some consequences of water quality degradation include higher sewage treatment costs (Dearmont et al. 1998), seafood price increases (e.g., Smith et al. 2017), and compromises to fish reproduction (Tuckey and Fabrizio 2016). There are links between nitrate in drinking water and birth defects [neural tube and spinal cord including spina bifida, oral cleft defects and limb deficiencies (Brender et al. 2013)], and bladder and thyroid cancer (Ward et al. 2018). Today’s agricultural and urban nutrient loadings are widely agreed as dominant controlling influences on the size and severity of coastal hypoxic zones that expanded dramatically throughout the world in the last 50 years (Rabalais et al. 2007; Díaz and Rosenberg 2008; Carstensen et al. 2014; Lefcheck et al. 2018). The strictly nutrient-related issues are co-developing with ocean acidification and climate change whose cumulative and synergistic interactions may be even more socially and ecologically significant (Moss et al. 2011). Plastics fill oceans (Lavers and Bond 2017), pharmaceuticals are distributed in sewage (Kasprzyk-Hordern et al. 2009), and Covid-19 virus and other viruses spread in partially treated sewerage wastes from aging septic tanks (Farkas et al. 2020), unconstrained wetland treatment systems with insufficient hydrologic controls, and overloaded treatment systems. The world’s population of inhabitable land will be down to 0.55 ha per person by 2100 at moderate population growth (Turner 2017), making disease transmittal more likely, not less likely, as distances between parasite and host are diminished.

It is far better to prevent damage first, of course, than fix later, and the surge in new chemicals added each year is daunting. Further, it is difficult to determine the severity of chronic effects when monitoring records are infrequent or difficult to obtain, which they often are. The promulgation and acceptance of the CWA and CAA demonstrate how public policy can change for the better for everyone who is demonstrably ‘downstream’ in a world of cycling pollutants.

## Supplementary Information

Below is the link to the electronic supplementary material.

Supplementary material 1 (PDF 223 kb)
