# Supplementary material for: Declining bacteria, lead, and sulphate, and rising pH and oxygen in the lower Mississippi River
Source: Ambio. 2021 Feb 7;50(9):1731–8. doi: 10.1007/s13280-020-01499-2 (PMC7868078; doi:10.1007/s13280-020-01499-2)
Supplement: Supplementary file 1 — Supplementary material 1 (PDF 223 kb) [file 13280_2020_1499_MOESM1_ESM.pdf]

Ambio:
Electronic Supplementary Material
This supplementary material has not been peer reviewed.

Declining bacteria, lead, and sulphate, and rising pH and oxygen in the lower Mississippi River

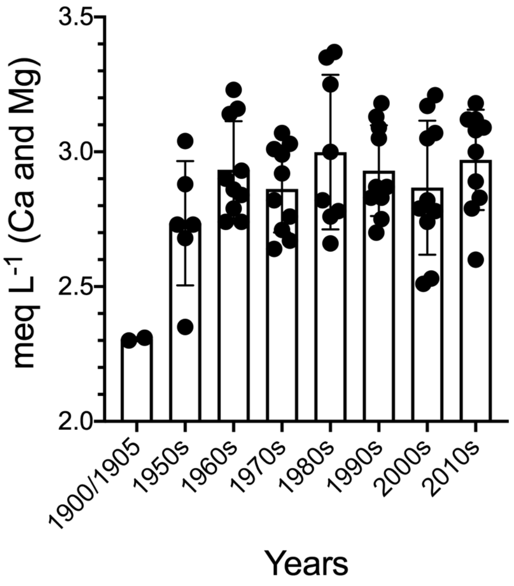

Figure S1. Annual Mg and Ca concentrations at St. Francisville, La, USA, from 1900 to 2019.

Table S1. Bacterial densities in the Mississippi River. A. Total coliforms and fecal coliform densities at St. Francisville, La (USGS). B. Total bacteria at New Orleans, La (NOSWB). C. Total coliforms at New Orleans, La (NOSWB). D. Total fecal coliforms at Belle Chasse, La (LaDEQ). The standard error of the mean (SEM) is given when the individual numbers were available.

A. Total coliforms and fecal coliform densities at St. Francisville, La (USGS)

	Total coliforms				Fecal coliforms		
	Average				Average		
Year	# / 100 ml	SEM	Count	Year	# / 100 ml	SEM	Count
1968	-	-	0	1968		-	0
1969	490	-	1	1969	150	-	1
1970	4583	3811	4	1970	275	109	4
1971	1100	-	1	1971	461	-	1
1972		-		1972		-	0
1973	3912	855	25	1973	1135	658	24
1974	1878	325	16	1974	322	63	15
1975	1727	614	6	1975	187	44	6
1976	2560	533	5	1976	220	68	5
1977	815	485	2	1977	863	670	3
1978	1877	446	6	1978	297	145	6
1979	4483	3839	4	1979	328	220	5
1980	748	88	5	1980	78	30	4
1981	310	121	4	1981	33	13	4
1982	2023	854	7	1982	364	240	7
1983	158	75	3	1983	22	12	3
1984	1123	592	3	1984	213	150	3
1985	627	209	6	1985	72	23	6
1986	970	178	8	1986	103	24	8
1987	582	125	10	1987	103	21	10
1988	513	210	5	1988	75	33	5
1989	909	225	7	1989	54	19	7
1990	796	233	5	1990	156	52	5
1991	1380	785	6	1991	152	48	6
1992	581	285	4	1992	50	13	4
1993	1000	352	5	1993	81	24	6
1994	260	40	2	1994	44	-	1
1995	288	145	3	1995	52	26	4
1996	385	365	2	1996	53	16	6
1997	436	164	8	1997	41	13	8

1998	476	122	8	1998	76	29	8
1999	134	62	4	1999	92	57	4
				2000	26	8	4
				2001	360	179	4
				2002	160	54	9
				2003	86	23	5
				2004	114	28	9
				2005	59	17	10
				2006	116	67	7
				2007	2	-	1
				2008	189	51	10
				2009	18	5	2

B. Total bacteria at New Orleans, La (NOSWB)

Year	Total bacteria Average # / 100 ml	SEM	Count
1909	181429	14869	7
1910	419583	64186.2	12
1911	245333	57680.2	12
1912	325000	48060.9	12
1913	213333	40044.2	12
1914	180000	9454.84	12
1915	185000	8024.58	12
1916	195833	7633.49	12
1917	183333	6666.67	12
1918	210000	17407.8	12
1919	171667	10859.5	12
1920	204167	14431.6	12
1921	225833	15689.1	12
1922	220000	9770.08	12
1923	252500	21181.9	12
1924	224167	23914.6	12
1925	169167	9727.99	12
1926	183333	14788.1	12
1927	161667	6376.25	12

1928	153333	3553.35	12
1929	158333	5618.33	12
1930	162500	5789.88	12
1931	229167	28456.7	12
1932	405000	44949.5	12
1933	580833	88261.5	12
1934	220167	14515.8	12
1935	417458	65159	12
1936	769875	163760	12
1937	1444417	455667	12
1938	1270167	374055	12
1939	1051042	270653	12
1940	520392	125213	12
1941	817275	167358	12
1942	1797000		
1943	836000		
1944	557000		
1945	4284000	648377	12
1946	4100033	864768	12
1947	7806700	2456080	12
1948	1.2E+07	2382694	12
1949	1.2E+07	2902967	12
1950	1.5E+07	3190961	12
1951	7102192	921253	12
1952	1688058	239193	12
1953	1467467	33388	12

C. Total coliforms at New Orleans, La (NOSWB).

Year	Average # / 100 ml	Count
1953	2834	-
1954	2803	-
1955	2803	-
1956	7055	-

1957	8897	-
1958	3116	-
1959	-	-
1960	3009	-
1961	3013	-
1962	2440	-
1963	1353	-
1964	1100	-
1965	2538	-
1966	2121	-
1967	2560	-
1968	2424	-
1969	2815	-
1970	2012	-
1971	2260	-
1972	4195	-
1973	5132	-
1974	8964	-
1975	4618	-
1976	4082	-
1977	2830	-
1978	3099	-
1979	4700	-
1980	2800	177
1981	2390	180
1982	3400	159
1983	6200	180
1984	4400	188
1985	3130	182
1986	3400	189
1987	3519	185
1988	3400	192
1989	2456	366
1990	1718	357
1991	1987	363
1992	1685	366
1993	1632	369
1994	1597	359
1995	1420	361
1996	1827	364
1997	1220	354
1998	915	360
1999	905	365

2000	1070	365
2001	1650	343
2002	1400	356
2003	1480	346
2004	965	342
2005	-	-
2006	965	342
2007	879	301
2008	1110	301
2009	880	334
2010	940	357
2011	680	359
2012	740	355
2013	610	365
2014	510	362
2015	600	382
2016	690	259
2017	600	225
2018	640	237
2019	540	240

D. Total fecal coliforms at Belle Chasse, La (LaDEQ).

Year	Average # / 100 ml	SEM	Count
1978	4886	970	16
1979	3197	758	19
1980	2647	487	14
1981	660	122	20
1982	1102	243	22
1983	1195	385	24
1984	636	123	24
1985	652	111	24
1986	697	125	22
1987	1119	218	16
1988	690	123	24
1989	614	110	24
1990	737	257	20
1991	456	115	9
1992	422	107	12
1993	338	92	12
1994	229	56	12

1995	248	54	12
1996	400	185	12
1997	331	132	12
1998	293	83	12
1999	194	70	12
2000	292	194	12
2001	298	86	12
2002	156	48	12
2003	103	40	12
2004	152	40	12
2005	163	30	10
2006	81	14	8
2007	203	50	13
2008	244	94	11
2009	45	18	8
2010	48	19	12
2011	34	8	9
2012	38	11	8
2013	55	10	6
2014	27	5	9
2015	27	7	9
2016	41	14	8
2017	61	24	9
2018	80	27	11
